# Child-To-Parent Violence: Which Parenting Style Is More Protective? A Study with Spanish Adolescents

**DOI:** 10.3390/ijerph16081320

**Published:** 2019-04-12

**Authors:** Cristian Suárez-Relinque, Gonzalo del Moral Arroyo, Celeste León-Moreno, Juan Evaristo Callejas Jerónimo

**Affiliations:** Department of Education and Social Psychology, Pablo de Olavide University, 41013 Seville, Spain; gmorar@upo.es (G.d.M.A.); cmleomor@upo.es (C.L.-M.); jevaristocallejas@gmail.com (J.E.C.J.)

**Keywords:** parenting styles, child-to-parent violence, adolescence

## Abstract

The link between parenting style and violent behavior during adolescence has become a relevant topic of research over the last few years. In order to deepen the understanding of this relationship, the aim of the present study was to examine what type of parenting style (authoritative, indulgent, authoritarian, and neglectful) is more protective against child-to-parent violence (CPV). A total of 2112 adolescents of both sexes participated in this study (50.2% men and 49.8% women), aged between 12 and 18 years (*M* = 14. 72, *SD* = 1.55). A multivariate factorial design (MANOVA, 4 × 2 × 3) was applied using parenting style, sex, and age group (12–14, 15–16, and 17–18 years) as independent variables and dimensions of CPV (physical and verbal aggression against the mother and father) as dependent variables. As shown in the results, the lowest scores on all the dimensions of CPV examined corresponded to the adolescents from indulgent families. Further, two interaction effects were observed between parenting style and age in verbal aggression against the mother and verbal aggression against the father. Regarding these effects, the adolescents from indulgent families obtained the lowest scores in two of the three age groups analyzed (12–14 years and 15–16 years). In the 17–18 years group, adolescents from authoritative families obtained similar but lower values than those coming from families with an indulgent style of parenting. These findings suggest that indulgent style is the most protective parenting style against CPV and also highlight the importance of affective warmth, emotional nurturance, and support giving in preventing CPV.

## 1. Introduction

Which parenting style of socialization is more protective against child-to-parent violence? With the main goal of answering this question, in this paper, we analyze the relationship between parenting style and child-to-parent violence (CPV), taking age and sex into account.

Parenting style can be defined as the emotional climate in which parents raise their children [1]. Traditionally, the relationship between parenting style and child adjustment has been analyzed following a two-dimensional orthogonal model of parental socialization. These two dimensions have usually been called demandingness and responsiveness, but in recent research, they have also been labeled as involvement/acceptance and strictness/supervision [2,3,4]. Other terms, such as assurance [5], warmth [6,7] or love [8], can be found in earlier research instead of involvement/acceptance. Moreover, labels such as domination, hostility, inflexibility, control, firmness or restriction were used in earlier studies with similar meanings to strictness/supervision [6,7,8,9].

According to this model of parental socialization, the strictness/supervision dimension refers to the extent to which parents use control and supervision, make maturity demands, and maintain an assertive position of authority with their children. The involvement/acceptance dimension represents the degree to which parents show affection to their children, give them support, and communicate by reasoning with them [3,10,11]. Based on these two orthogonal dimensions, four parenting styles have been identified: (1) Authoritative (high strictness/supervision and high acceptance/involvement); (2) authoritarian (high strictness/supervision and low acceptance/involvement); (3) indulgent (low strictness/supervision and high acceptance/involvement); and (4) neglectful (low strictness/supervision and low acceptance/involvement) [12,13,14,15,16].

On the other hand, child-to-parent violence (CPV) is defined in the scientific literature as any repeated act of abuse (physical, psychological or economic), committed by children towards parents (or any other figure that occupies the role of authority in the family) [17,18,19,20].

With respect to the information available on the prevalence of CPV, studies conducted in different countries identify rates ranging from 4.6% to 22% for physical aggression perpetrated at least once within a year by adolescents from 12–18 years (e.g., [21,22,23,24,25,26,27,28,29]). The highest CPV levels are usually detected in the case of verbal violence with rates ranging from 45% to 90% [22,28,30,31,32,33,34]. In addition, an important aspect that can be observed in the results of studies carried out not only in Spain but also in other countries is that mothers are chosen more frequently than fathers as targets of the aggression (e.g., [23,24,28,31,35,36,37,38,39,40]), although no significant differences have been observed in the case of the most severe forms of aggression [30,36].

Regarding the relationship between parenting style of socialization and CPV, studies have often linked CPV with authoritarian style of parenting (characterized by the exercise of a great control over minors and even the regular use of corporal punishment) [21,41,42,43,44,45]. However, after reviewing recent research, it is observed that adolescent aggressors come largely from family contexts where indulgent or neglectful styles prevail, i.e., homes where violent behavior is tolerated and aggression is often favored and reinforced [21,38,42,46,47]. In this sense, neglectful style has been associated with a high probability of physical and verbal aggression against parents. Another important aspect to be considered when analyzing CPV is that neglectful style of parenting is more commonly exercised by fathers, while indulgent style is used more frequently by mothers [31,38,48,49]. Finally, we can also find some studies claiming that violent behavior in adolescents (including CPV) is mainly related to the indulgent style of parental socialization [46,50,51].

It is important to point out that in many of the studies carried out in the last decades, authoritative style has been identified as the main protective style against CPV, and has also been connected to the optimal results for a broad range of child outcomes, such as psychosocial development, school competence, self-esteem or self-reliance [52,53,54,55]. However, despite the relevance of these results, they should be interpreted with caution due to the fact that most of the studies regarding parenting style of socialization have been conducted in the Anglo-Saxon context. In addition, it is important to highlight the results from several recent studies that show affective warmth, emotional nurturance, and support as the most relevant protective factors against CPV, even in the cases of family contexts where limits were not clearly established by parents [43,56,57,58,59]. Indeed, indulgent parenting style has been connected in several studies to equal or even better optimal development than the authoritative style in developmental outcomes such as self-esteem [60], internalization of values [58], psychosocial maturity [61] or academic competence [62]. Additionally, in order to prevent adolescent violence and other externalized behaviors, indulgent parenting offers a broad protection against antisocial tendency [61], behavioral problems and school misconduct [62], drug use [63] or CPV [43,56,57,58,59]. Even the authoritarian parenting style seems to be protective for some outcomes, as suggest studies from United States with ethnic minorities [64,65] or from Arab countries [66].

### 1.1. Sex and Age of the Aggressor

Most of the studies reveal that the majority of offenders are males [24,39,40,41,67]. However, we can find mixed results in respect of sex-related differences in the perpetration of CPV. For example, some studies indicate higher rates in boys than in girls for all types of CPV [68,69], but others suggest that verbal aggression is more frequent in girls than in boys, whereas physical aggression is more used by boys [28,30,70,71]. Part of the explanation for these mixed outcomes might be found in the type of methodology used in each research when analyzing CPV. In this sense, although most of the existing data relating to CPV proceed from studies that use community samples, some research works conducted using clinical samples can also be found [72].

The age range for CPV prevalence could be established between 4 and 24 years, but most of the detected cases take place in mid-adolescence (14–17 years) and then gradually decline as age increases [24,43,49,73,74].

Finally, very few studies in scientific literature have addressed the relationship between age and sex of the aggressor. Mahoney, O’Donnelly, Lewis, and Maynard affirm that with age, regardless of the sex of the child, aggressions towards fathers are more frequent than against mothers [75]. Further, Walsh and Krienert find that as age increases, the prevalence of CPV is higher in boys than in girls, perhaps because of the late maturation (physically and psychologically) in boys [69]. On the other hand, girls begin to attack their parents at a younger age than boys, maybe as a result of early female maturation, although the severity level of the assault seems to decrease with age in the case of girls, while it increases in boys [69,76,77].

### 1.2. The Present Study

In the present study, we analyze the relationship between parenting style of socialization and child-to-parent violence (physical and verbal aggression against the father and mother), considering the sex and age of the adolescent. The main goal we propose is to obtain a valid and relevant answer to the following question: Which parenting style is more protective against CPV? In this sense, we must highlight the fact that recent studies have examined some of the aspects analyzed in our study and have provided relevant information in the field of CPV, but without addressing the specific objective we propose. For example: Calvete, Orue, Gamez-Guadix, and Bushman (2015) [78] analyzed the relationship between parenting style and CPV using two dimensions of parenting (parental warmth and permissive parenting); Calvete et al. (2015) [79] conducted a qualitative research to examine the extent to which being exposed to family violence and parental discipline influences the development of CPV; Del Hoyo-Bilbao, Gámez-Guadix, and Calvete (2018) [80] examined CPV prevalence and age-and sex-related differences in a sample of adolescents; Lyons, Bell, Fréchette, and Romano (2015) [34] investigated the influence of family violence on CPV by focusing on a range of child disciplinary practices and some macro-level constructs, including exposure to community violence; Gámez-Guadix, Jaureguizar, Almendros, and Carrobles (2012) [81] examined the relationship between parenting style and CPV using a sample of Spanish university students; Moreno-Ruiz, Estévez, Jiménez, and Murgui (2018) [82] presented a work which focused on the relationship between parenting style and reactive and proactive school violence among peers. In this last case, both parenting style and the possible interaction between all variables (sex, age, and style of parenting) were included in the analysis but using a different dependent variable (school violence among peers).

In conclusion, all these previous works are suggestive and interesting, but none of them do a specific analysis of the relationship between parenting styles and CPV by sex and age similar to the one that is proposed in the present study. More specifically, based on the information collected from recent studies, we researched three hypotheses:

(1) Adolescents from families with an authoritative and indulgent parenting style will obtain lower scores on all dimensions of CPV (physical aggression against the mother, physical aggression against the father, verbal aggression against the mother, verbal aggression against the father) than those coming from authoritarian and neglectful families;
(2) Boys will show the highest levels of CPV in physical violence against the father and mother, and girls will obtain the highest scores in the case of verbal violence towards father and mother;
(3) Adolescents in the age group that corresponds to middle adolescence (15–16 years) will obtain higher scores on all dimensions of CPV than those in the age groups 12–14 and 17–18 years.

## 2. Materials and Methods

### 2.1. Participants

A total of 2119 adolescents participated in this study. Adolescents were selected from 9 public and private secondary schools located in the region of Andalusia (Spain). The selection of the participants was carried out through a stratified random sampling. The sampling units were geographical area (urban or rural) and school ownership (public or private). Statistical analyses showed no significant mean differences in the dependent variables as a result of the specific location of the school and the type of school ownership. Missing data were treated using the listwise deletion procedure (i.e., records which contained any missing value were not included in the analysis), and a total of 7 cases were deleted from the original sample. The final sample consisted of 2112 adolescents of both sexes (50.2% men and 49.8% women), aged between 12 and 18 years (*M* = 14. 72, *SD* = 1.55).

### 2.2. Procedure

Once the educational centers were selected and their management school teams confirmed their willingness to participate in the study, the researchers arranged a meeting to apply for the corresponding permits and explain the objectives, specific procedure, and scope of the investigation to the school management and teachers. Subsequently, the researchers asked for the voluntary collaboration of the students and sent a letter to the parents of those adolescents who expressed their willingness to collaborate in the study, in order to obtain a written consent from their families.

After obtaining the required permits, the instrument was administrated on the agreed date. The administration of the questionnaire was carried out by a group of expert and trained researchers in the adolescents’ usual classrooms during a regular period of class (which lasted approximately 50 min). The students were informed that their participation in the study was voluntary and anonymous and also that they had the option to leave the session anytime they wanted without filling out the questionnaire. Finally, it is important to underline that this research was conducted according to the fundamental principles included in the Declaration of Helsinki and its subsequent updates and was also approved by the Ethics Committee of the Pablo de Olavide University of Seville.

### 2.3. Materials

Two scales were used to obtain the information needed for the purpose of the study: Parental socialization scale (ESPA29) and conflict tactics scales (CTS2)—child-to-parent version. 

Parental Socialization Scale (ESPA29) [3,14,15,83]. This scale consists of 212 items and was developed to analyze which parenting style of socialization is used by parents when educating their children. Its design is based on the two-dimensional theoretical model of parental socialization [1,2]. The adolescent evaluates his/ her parents’ behavior (mother and father independently) in 29 everyday family life situations. Thirteen of these situations are negative ones, that is to say, situations where the adolescent goes against the family rules (for example, “If I fight with a friend or one of my neighbors”). Then, the adolescent has to indicate, on a scale ranging from 1 (never ) to 4 (always), the degree to which his/her father or mother responds to those situations on the basis of verbal coercion (“He/She…Scolds me”), dialogue (“He/She…Talks to me”), displeasure (“He/She…Doesn’t care”), physical coercion (“He/She…Hits me”), and deprivation (“He/She…Deprives me of something”). The other 16 situations are positive ones (i.e., situations in which the adolescent behavior follows the family rules). In those cases, the adolescent has to indicate the degree to which his/ her father or mother shows affection (“He/She…Shows me love”) and indifference (“He/She…Is indifferent”). In the aggregate, the adolescent responds to 212 items: 106 regarding his/her father’s behavior and 106 responses in respect of his/her mother’s behavior. From those responses, a global measure for each parent is obtained in the two dimensions: Involvement/acceptance and strictness/supervision. This global measure is then used for classifying the parenting style of each parent as authoritative, indulgent, authoritarian or neglectful.

The score on the dimension acceptance/involvement is obtained by averaging the scores shown in the subscales of affection, dialogue, indifference, and displeasure (the subscales of indifference and displeasure are inverted due to the fact that they are inversely related to the dimension). The score on the strictness/supervision dimension is calculated by averaging the scores observed in the subscales of verbal coercion, physical coercion, and deprivation. In this study, the Cronbach alpha reliability coefficients for the scale were: Acceptance/involvement 0.89; and strictness/supervision 0.94; and for the seven subscales, we obtained the following coefficients: Affection 0.95; indifference 0.96; dialogue 0.95; displeasure 0.92; verbal coercion 0.95; physical coercion 0.94; and deprivation 0.94.

With respect to the parenting typology, according to the procedure suggested in previous studies [14,61,77], families were classified as follows: Authoritative, families who scored above the 50th percentile on both acceptance/involvement and strictness/imposition dimensions; Neglectful, families who scored below the 50th percentile on both dimensions; Authoritarian, families who scored above the 50th percentile on strictness/imposition and below the 50th percentile on acceptance/involvement; Indulgent, families who scored above the 50th percentile on acceptance/involvement and below the 50th percentile on strictness/imposition.

Conflict Tactics Scales (CTS2) [42,81,84]. The original scale is composed of 6 items measuring two dimensions: Verbal aggression (i.e., “I shouted or yelled at my parents”) and physical aggression (i.e., “I hit my parents with something that could hurt”). The items are rated on a Likert five-point scale, ranging from 0 = never to 4 = many times. To obtain the information required from the child about both parents, he/she has to respond two times for each item of the CTS scale (one for mother and one for father). The Cronbach alpha reliability coefficients in the present study were 0.80 for the verbal aggression subscale and 0.71 for the physical aggression subscale.

## 3. Results

Statistical analysis in the present study was carried out using SPSS software (version 20.0; IBM, Armonk, NY, USA). First, we calculated the cross-distribution of parenting styles with sex and age groups (see Table 1).

Second, we applied a multivariate factorial design (MANOVA, 4 × 2 × 3) with the dimensions of CPV (physical aggression against the mother, physical aggression against the father, verbal aggression against the mother, verbal aggression against the father) as dependent variables, and considering the parenting style (authoritative, indulgent, authoritarian, and neglectful), sex (male and female), and age group (12–14 years, 15–16 years, and 17–18 years) as independent variables. 

As shown in Table 2, the multivariate analysis of variance showed statistically significant main effects for parenting style, Λ = 0.98, *F*(12, 5516.7) = 4.089, *p* < 0.001, sex, Λ = 0.99, *F*(4, 2085) = 5.920, *p* < 0.001, and age, Λ = 0.97, *F*(8, 4170) = 7.506, *p* < 0.001. Moreover, a statistically significant interaction for parenting style and age, Λ = 0.98, *F*(24, 7274.9) = 1.630, *p* < 0.05, was obtained.

### 3.1. Parenting Style and CPV

With respect to the variable parenting style, we observed significant main effects on physical aggression against the mother, *F*(3, 2108) = 6.83, *p* < 0.001, physical-aggression against father, *F*(3, 2108) = 4.05, *p* < 0.01, verbal-aggression against mother, *F*(3, 2108) = 27.99, *p* < 0.001, and verbal-aggression against father, *F*(3, 2108) = 19.56, *p* < 0.001. The highest scores on all the types of violence discussed corresponded to adolescents who came from family contexts with authoritarian styles, followed by those from neglectful families. The lowest values in all types of violence were obtained by adolescents from families that developed an indulgent style of parenting (see Table 3).

Further, two interaction effects were observed for parenting style and age on verbal aggression against the mother, *F*(6, 2088) = 2.645, *p* <0.05, and verbal-aggression against father, *F*(6, 2088) = 3.422, *p* < 0.01, (see Table 4). On the other hand, no interaction effect for parenting style and sex was detected.

The highest scores on verbal aggression against the mother and verbal aggression against the father corresponded to adolescents aged 15–16 years who came from family contexts with authoritarian styles. Indeed, the authoritarian style obtained higher values on verbal aggression against the mother and against the father than the rest of the parenting styles examined regarding all age groups, with the only exception of the scores registered in the 17–18 years group on verbal aggression against the mother. In this case, adolescents aged 17–18 years from neglectful families scored higher than those at the same age coming from other family contexts (although similar scores were observed in the authoritarian style group). 

**Figure 1:**
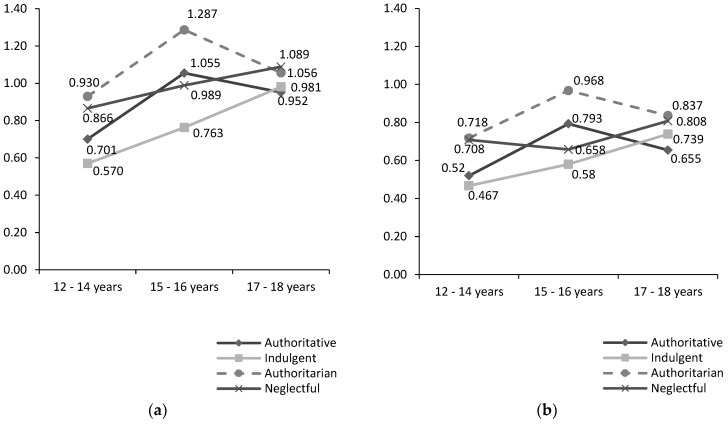
Means of parenting styles by age for verbal aggression against mother (**a**) and father (**b**).

On the other hand, the lowest values in both dimensions of CPV were obtained by adolescents aged 12–14 years from families that develop an indulgent style of parenting. Similar to what was observed in respect of the highest scores, indulgent style obtained lower values than the rest of the parenting styles analyzed in the 12–14 years and 15–16 years age groups; however, adolescents from authoritative families showed the lowest scores on both verbal aggression against the mother and verbal aggression against the father in the 17–18 years group (see Figure 1a,b). 

### 3.2. Sex-Related Differences in CPV

As Table 5 presents, girls obtained higher mean values than boys in verbal aggression against the mother, *F*(1, 2110) = 12.39, *p* < 0.001, and verbal-aggression against father, *F*(1, 2110) = 7.73, *p* < 0.01. We also observed a main effect for sex on physical-aggression against father, *F*(1, 2110) = 9.40, *p* < 0.01, but in this case, boys obtained higher scores than girls. On the other hand, no significant effect for sex upon physical aggression against the mother was detected.

### 3.3. Age-Related Differences in CPV

We observed a statistically significant main effect for age upon CPV only in the case of verbal aggression. No significant effect for age on physical aggression (against the father and mother) was detected. 

Adolescents aged 17–18 years obtained the highest scores in the case of verbal aggression against the mother, *F*(2, 2109) = 40.41; *p* < 0.001, and verbal aggression against the father, *F*(2, 2109) = 20.21, *p* < 0.001). On the other hand, the lowest values in all types of violence were obtained by adolescents from 12 to 14 years (see Table 6).

## 4. Discussion

The main goal of this study was to examine which parenting style is more protective against child-to-parent violence (CPV). To achieve that objective, we analyzed the relationship between parenting style and CPV in a sample of Spanish adolescents, taking sex and age into account. 

First, we detected a significant main effect for parenting style on CPV. We observed that the highest scores on all dimensions of CPV were obtained by adolescents from authoritarian families, followed by those who came from family contexts with a neglectful style of parenting. These findings are consistent with previous research which indicates that the styles which correlate positively with CPV are, generally the ones based mainly on coercive strategies but also those based on lack of monitoring and low control over the child’s behavior [26,44,80,85].

Regarding the influence of monitoring and control on CPV, we must underline the differences observed in the scores obtained by the neglectful and the indulgent style, respectively. Although these two styles of parenting use a low level of strictness/supervision in their educative practices, the neglectful style showed high levels of CPV, while the indulgent style obtained the lowest scores on all the dimensions examined. In addition, the adolescents of the study who came from family contexts with an authoritative style obtained lower scores than those coming from homes where authoritarian or neglectful styles were developed. These results confirm our first hypothesis and are consistent with findings observed in previous research which indicate the protective effect of developing educative practices based on affective warmth and support in preventing CPV [56,57,58,78,84,86].

Secondly, we found a significant interaction for parenting style and age that should be highlighted because it may represent one of the most interesting contributions of our present work. Specifically, two interaction effects were obtained: The first one was observed on verbal aggression against the mother, and the second one was found on verbal aggression against the father.

The information provided by these interactions is especially interesting because it points out the importance of considering the age of the adolescent when analyzing the influence of parenting style on CPV. In this sense, one aspect that deserves to be highlighted is the fact that the adolescents from indulgent families obtained the lowest scores in our study on both verbal aggression against the mother and verbal aggression against the father in two of the three age groups analyzed (12–14 years and 15–16 years). Only in the 17–18 years group did the authoritative style obtain lower values than the indulgent style. Second, the differences observed between the lowest scores on these dimensions (obtained by the indulgent style) and the highest ones (showed by the authoritarian style) are significant in the case of the adolescents from 12 to 16 years. However, these differences are much smaller in the group of 17–18 years. Finally, the authoritarian style seems to be the best predictor of verbal aggression against both the father and mother in the adolescents from 12 to 18 years. The only exception to this was observed in the case of verbal aggression against the mother in adolescents aged 17–18 years, where the neglectful style showed similar but higher scores than the authoritarian style of parenting.

With respect to the variable sex, we observed a significant main effect for sex on verbal aggression towards parents. As shown in the results, girls obtained the highest scores on verbal aggression against both the father and mother. We also observed a significant main effect for sex upon physical-violence against the father but, in this case, boys showed the highest scores. These findings confirm our second hypothesis and are supported by most previous research which says that verbal aggression is used more by girls than by boys, and physical aggression is more frequent in boys [21,28,29,70,71]. 

However, our results should be interpreted with caution due to the fact that we can also find some studies showing no significant differences in rates of perpetration between boys and girls [28,43,71,79] or even finding higher rates in boys than in girls in all types of CPV [68,69]. A possible explanation for these mixed results may be found in the method used by each study for gathering information. For example, results can show some variations from one study to another depending on the type of sample used (community or clinical sample) [72]. On the other hand, according to some authors, girls tend to report greater levels of trivial violence (e.g., shouting at a parent once) than boys [30,49,87]. Taking this into account, maybe the studies that use a low threshold for measuring verbal abuse could be increasing the scores obtained by girls compared to those shown by boys. Moreover, the cultural or macrosystemic factors that affect adolescent should be included when interpreting the results. For example, girls can feel a greater social pressure than boys and also feel more embarrassed when they have to report any physical aggression towards parents. According to this, the real prevalence of physical aggression perpetrated by girls could be underestimated.

In the third hypothesis of our study, we predicted that the highest values obtained in the different CPV dimensions analyzed would be observed in the 15–16 years group (mid-adolescence). First, no significant main effect for age on physical violence was detected, but we observed a significant effect for age on verbal violence against parents (father and mother). On both dimensions, the highest scores corresponded to subjects aged 17–18 years. Strictly speaking, our hypothesis has been rebutted. However, we think it is important when interpreting these results to take into account that the scores obtained by the 15–16 years group and those observed in the 17–18 years group were very similar. Then, we could say that these results point (at least partially) in the direction suggested by previous research which indicates that CPV reaches a peak in mid-adolescence (14–17 years) and then gradually declines with age [24,29,39,49].

Finally, we want to underline that no significant interaction for age and sex was found in the present study. In this case, the results are consistent with a large part of the existing literature, but we can also find some studies that show different outcomes. For example, according to the results observed in recent works, as age of adolescents increases, fathers are more likely to be the targets of the aggression by both boys and girls, although no significant differences have been detected between boys and girls in respect of the chosen target (mother or father) in the case of most severe forms of aggression [25,47,69,87]. However, the severity of the assault is generally higher in boys than in girls aged 16 to 18 years [75,76,77].

To sum up, the findings of our study strengthen the idea defended by some authors regarding the importance of developing parental practices based on affectivity, parental warmth, and support for preventing violent behaviors in adolescence. These practices are included in the involvement/acceptance dimension and are associated with the indulgent and authoritative styles of parenting. In this sense, the results obtained in the present study point out the indulgent style as the most protective parenting style against CPV.

On the other hand, we would like to indicate some limitations regarding this study. First, the results obtained in our research are based on the opinion of the adolescents. We think that for future studies, a deeper analysis of the relationship between parenting style and child-to-parent violence could be achieved if the view of children’s parents were also included. Second, the “family structure” variable (i.e., married parents, divorced parents, stepparents, etc.) has not been considered in our research, so we have not measured its possible effect on the relationship between parenting style and CPV. Third, we used a quantitative strategy in our study, but when working with a sample of adolescents to examine CPV, the inclusion of qualitative methodology (together with quantitative methods) may provide relevant information for the explanation and description of the phenomenon, mainly because the analysis of adolescents’ speeches allows researchers to explore the implicit theories held by boys and girls regarding CPV [82,88,89]. 

However, despite the cited limitations, we would like to highlight that this study provides some interesting and valuable information, which is especially relevant for the development of future research work, as well as for the design and implementation of intervention and guidance programs in the field of parental education.

## 5. Conclusions

Findings obtained in our research provide interesting information about the relationship between parenting style and CPV. First, results pointed in the expected direction regarding the significant relationship between the authoritarian and neglectful parental styles and occurrence of CPV during adolescence [21,38,43,45,46,49]. Second, we must highlight, perhaps as the main contribution of the present study, the results obtained in respect of the interaction between parenting style, CPV, and age of the adolescent. In this sense, the adolescents from authoritarian families obtained the highest scores on verbal aggression against the father in the three age groups analyzed (ranging from 12 to 18 years). Further, the authoritarian style showed the highest values on verbal aggression against the mother in the participants aged 12–14 and 15–16 years; however, the neglectful style showed higher levels in the 17–18 years group. 

On the other hand, the lowest values observed on all dimensions of CPV corresponded to the adolescents who came from families that developed an indulgent style of parenting. In respect of the specific relationship between parenting style, CPV, and age, we observed that adolescents from indulgent families obtained the lowest scores on both verbal aggression against the mother and verbal aggression against the father in two of the three age groups analyzed (12–14 years and 15–16 years). Only in the 17–18 years group did the authoritative style obtain lower values than the indulgent style.

These results are consistent with the findings showed in recent research (see References [43,57,58,59,78]) and point in a different direction with respect to what is suggested in some other works in which indulgent style of parenting has been identified as a risk factor for the development of violent behavior towards parents by adolescents [52,53,54,55]. Moreover, findings of the present study indicate the importance of taking the age variable into account when studying the relationship between parenting style and CPV. Finally, in order to respond to the relevant question that has guided our research, according to the results obtained, the indulgent style could be considered as the most protective parenting style of socialization against CPV.

## Figures and Tables

**Table 1:** Sociodemographic variables.

Variables	Total Sample *N* (%)	Parenting Style			
		Neglectful (*n* = 568) *N* (%)	Authoritarian (*n* = 546) *N* (%)	Indulgent *(n* = 417*)* *N* (%)	Authoritative (*n* = 581) *N* (%)
Sex					
Boys	1061 (50.2%)	299 (28.2%)	303 (28.6%)	172 (16.2%)	287 (27%)
Girls	1051 (49.8%)	269 (25.6%)	243 (23.1%)	245 (23.3%)	294 (27%)
Age Group					
(12–14)	980 (46.4%)	155 (15.8%)	237 (24.2%)	210 (21.4%)	378 (38.6%)
(15–16)	808 (38.3%)	260 (32.2%)	219 (27.1%)	154 (19.1%)	175 (21.7%)
(17–18)	324 (15.3%)	153 (47.2%)	90 (27.8%)	53 (16.4%)	28 (8.6%)
Total	2112 (100%)	568 (26.9%)	546 (25.9%)	417 (19.7%)	581 (27.5%)

**Table 2:** MANOVA results for all the studied variables (4 ^a^ × 2 ^b^ × 3 ^c^).

Source of Variation	*Λ*	*F*	*dfbetween*	*Dferror*	*p*	*η* ^2^
(A) Parenting Style ^a^	0.977	4.089	12	5516.683	<0.001 ***	0.008
(B) Sex ^b^	0.989	5.920	4	2085	<0.001 ***	0.011
(C) Age ^c^	0.972	7.506	8	4170	<0.001 ***	0.014
A × B	0.994	0.978	12	5516.683	0.467	0.002
A × C	0.981	1.630	24	7274.906	<0.05 *	0.005
B × C	0.997	0.670	8	4170	0.719	0.001
A × B × C	0.989	1.003	24	7274.906	0.457	0.003

a_1_, Neglectful, a_2_, Authoritarian, a_3_, Indulgent, a_4_, Authoritative; b_1_, Boy, b_2_, Girl.; c_1_, 12–14 years, c_2_, 15–16 years, c_3_, 17–18 years. *** *p* < 0.001; * *p* < 0.05.

**Table 3:** Means, standard deviations, and differences between parenting styles and dimensions of child-to-parent violence (CPV).

Type of CPV		Parenting Style					
		Authoritative	Indulgent	Authoritarian	Neglectful	*F*(3, 2108)	*η* ^2^
Physical aggression	against mother	0.038 (0.186) ^b^	0.014(0.073) ^b^	0.068 (0.209) ^a^	0.050 (0.226) ^a^	6.834 ***	0.01
	against father	0.024 (0.119)	0.012 (0.071) ^b^	0.045 (0.189) ^a^	0.038 (0.211)	4.053 **	0.006
Verbal aggression	against mother	0.820 (0.775) ^b^	0.694 (0.648) ^c^	1.094 (0.714) ^a^	0.982 (0.780) ^a^	27.990 ***	0.038
	against father	0.608 (0.665) ^c^	0.543 (0.569) ^d^	0.838 (0.695) ^a^	0.712 (0.656) ^b^	19.562 ***	0.027

Note. *** *p* < 0.001; ** *p* < 0.01; a > b > c > d.

**Table 4:** Means, standard deviations, and post-hoc comparisons between parenting styles, age, and dimensions of CPV (VAM, VAF).

CPV	Age	Parenting Style				*F*(6, 2088)	*η* ^2^	*Post-Hoc*
		Authoritative	Indulgent	Authoritarian	Neglectful			
VAM	12–14	0.701 (0.719) ^c^_1_	0.570 (0.611) ^c^_2_	0.930 (0.647) ^b^_2_	0.866 (0.733) ^b^_3_	2.645 *	0.008	a_1_ > b_1_, b_2_ > c_1_, c_2_ a_1_ > b_3_ > c_2_ a_1_, a_2_, a_4_ > b_4_ a_2_, a_3_, a_4_, a_5_ > c_1_, c_2_
	15–16	1.055 (0.844) ^a^_4_	0.763 (0.624) ^b^_4_	1.287 (0.784) ^a^_1_	0.989 (0.745) ^b^_1_			
	17–18	0.952 (0.735)	0.981 (0.747) ^a^_5_	1.056 (0.583) ^a^_3_	1.089 (0.807) ^a^_2_			
VAF	12–14	0.520 (0.602) ^c^_1_	0.467 (0.548) ^c^_2_	0.718 (0.603) ^b^_1_	0.708 (0.712) ^b^_2_	3.422 **	0.007	a_1_ > b_1_ > c_1_, c_2_ a_1_ > b_2_ > c_2_ a_1_ > b_3_, b_4_ a_2_, a_3_, a_4_ > c_1_, c_2_
	15–16	0.793 (0.744) ^a^_4_	0.580 (0.556) ^b^_4_	0.968 (0.799) ^a^_1_	0.658 (0.582) ^b^_3_			
	17–18	0.655 (0.729)	0.739 (0.634)	0.837 (0.594) ^a^_2_	0.808 (0.708) ^a^_3_			

Note: VAM = Verbal aggression against mother; VAF = Verbal aggression against father. ** *p* < 0.01; * *p* < 0.05.

**Table 5:** Means, Standard deviations and differences between sex and dimensions of CPV.

Type of CPV		Sex		*F*(1, 2110)	*η* ^2^
		Boy	Girl		
Physical aggression	against mother	0.053 (0.208)	0.036 (0.169)	3.541	0.001
	against father	0.042 (0.201)	0.020 (0.108)	9.402 **	0.003
Verbal aggression	against mother	0.852 (0.736)	0.967 (0.763)	12.387 ***	0.006
	against father	0.643 (0.654)	0.723 (0.666)	7.732 **	0.004

Note. *** *p* < 0.001; ** *p* < 0.01.

**Table 6:** Means, standard deviations, and differences between age and dimensions of CPV.

Type of CPV		Age Group			*F*(2, 2109)	*η* ^2^
		12–14 years	15–16 years	17–18 years		
Physical aggression	against mother	0.037 (0.181)	0.051 (0.196)	0.050 (0.197)	1.409	0.001
	against father	0.024 (0.145)	0.037 (0.177)	0.035 (0.170)	1.438	0.001
Verbal aggression	against mother	0.754 (0.694) ^b^	1.041 (0.789) ^a^	1.051 (0.733) ^a^	40.412 ***	0.037
	against father	0.586 (0.618) ^b^	0.756 (0.693) ^a^	0.791 (0.667) ^a^	20.214 ***	0.019

Note. *** *p* < 0.001; a > b.
